# 2-Hydroxypropyl-β-Cyclodextrin Acts as a Novel Anticancer Agent

**DOI:** 10.1371/journal.pone.0141946

**Published:** 2015-11-04

**Authors:** Masako Yokoo, Yasushi Kubota, Keiichi Motoyama, Taishi Higashi, Masatoshi Taniyoshi, Hiroko Tokumaru, Rena Nishiyama, Yoko Tabe, Sakiko Mochinaga, Akemi Sato, Naoko Sueoka-Aragane, Eisaburo Sueoka, Hidetoshi Arima, Tetsumi Irie, Shinya Kimura

**Affiliations:** 1 Division of Hematology, Respiratory Medicine and Oncology, Department of Internal Medicine, Faculty of Medicine, Saga University, Saga, Japan; 2 Department of Transfusion Medicine, Saga University Hospital, Saga, Japan; 3 Graduate School of Pharmaceutical Sciences, Kumamoto University, Kumamoto, Japan; 4 Department of Clinical Laboratory Medicine, Juntendo University School of Medicine, Tokyo, Japan; 5 Department of Pharmacy, Saga University Hospital, Saga, Japan; 6 Department of Clinical Laboratory Medicine, Faculty of Medicine, Saga University, Saga, Japan; 7 Program for Leading Graduate Schools “HIGO (Health life science: Interdisciplinary and Global Oriented) Program”, Kumamoto University, Kumamoto, Japan; B.C. Cancer Agency, CANADA

## Abstract

2-Hydroxypropyl-β-cyclodextrin (HP-β-CyD) is a cyclic oligosaccharide that is widely used as an enabling excipient in pharmaceutical formulations, but also as a cholesterol modifier. HP-β-CyD has recently been approved for the treatment of Niemann-Pick Type C disease, a lysosomal lipid storage disorder, and is used in clinical practice. Since cholesterol accumulation and/or dysregulated cholesterol metabolism has been described in various malignancies, including leukemia, we hypothesized that HP-β-CyD itself might have anticancer effects. This study provides evidence that HP-β-CyD inhibits leukemic cell proliferation at physiologically available doses. First, we identified the potency of HP-β-CyD *in vitro* against various leukemic cell lines derived from acute myeloid leukemia (AML), acute lymphoblastic leukemia and chronic myeloid leukemia (CML). HP-β-CyD treatment reduced intracellular cholesterol resulting in significant leukemic cell growth inhibition through G_2_/M cell-cycle arrest and apoptosis. Intraperitoneal injection of HP-β-CyD significantly improved survival in leukemia mouse models. Importantly, HP-β-CyD also showed anticancer effects against CML cells expressing a T315I BCR-ABL mutation (that confers resistance to most ABL tyrosine kinase inhibitors), and hypoxia-adapted CML cells that have characteristics of leukemic stem cells. In addition, colony forming ability of human primary AML and CML cells was inhibited by HP-β-CyD. Systemic administration of HP-β-CyD to mice had no significant adverse effects. These data suggest that HP-β-CyD is a promising anticancer agent regardless of disease or cellular characteristics.

## Introduction

Advances in molecular targeting technologies have revolutionized cancer therapeutics, including imatinib mesylate for chronic myeloid leukemia (CML) and gefitinib for lung cancer [1,2]. Molecular-targeted drugs have superior anticancer effects compared to those of conventional chemotherapeutic agents, and have less adverse effects. However, resistance to chemotherapy, such as that resulting from point mutations or the existence of cancer stem cells, still hinders the treatment of cancer patients [3,4]. Thus, alternative therapeutic approaches that enhance neoplastic cell death are required for successful cancer treatment.

Cholesterol is one of the main components of lipid rafts, which provide signaling platforms capable of activating various cellular signaling pathways [5,6]. Cholesterol accumulation and/or dysregulated cholesterol metabolism is reported in various malignancies, including leukemia [7–9]. For example, breast and prostate cancer cell lines contain elevated levels of cholesterol, and are more sensitive to cholesterol depletion-induced cell death than their normal counterparts [10,11]. Breast cancer cells treated with mevalonate, a cholesterol precursor, demonstrate increased tumor cell growth *in vivo* and increased proliferation by accelerating cell-cycle progression [12]. Freshly isolated acute myeloid leukemia (AML) and CML cells show high rates of cholesterol import and/or synthesis [13,14]. Drug-resistant myeloid leukemia cell lines exhibit higher levels of 3-hydroxy-3-methylglutaryl coenzyme A (HMG-CoA) reductase, a rate limiting enzyme of the mevalonate pathway, suggesting that high cellular cholesterol may also improve leukemia cell survival and impart relative resistance to chemotherapy [14,15]. Taken together, modulation of cholesterol homeostasis might be a rational target for the development of anticancer agents.

**Fig 1:**
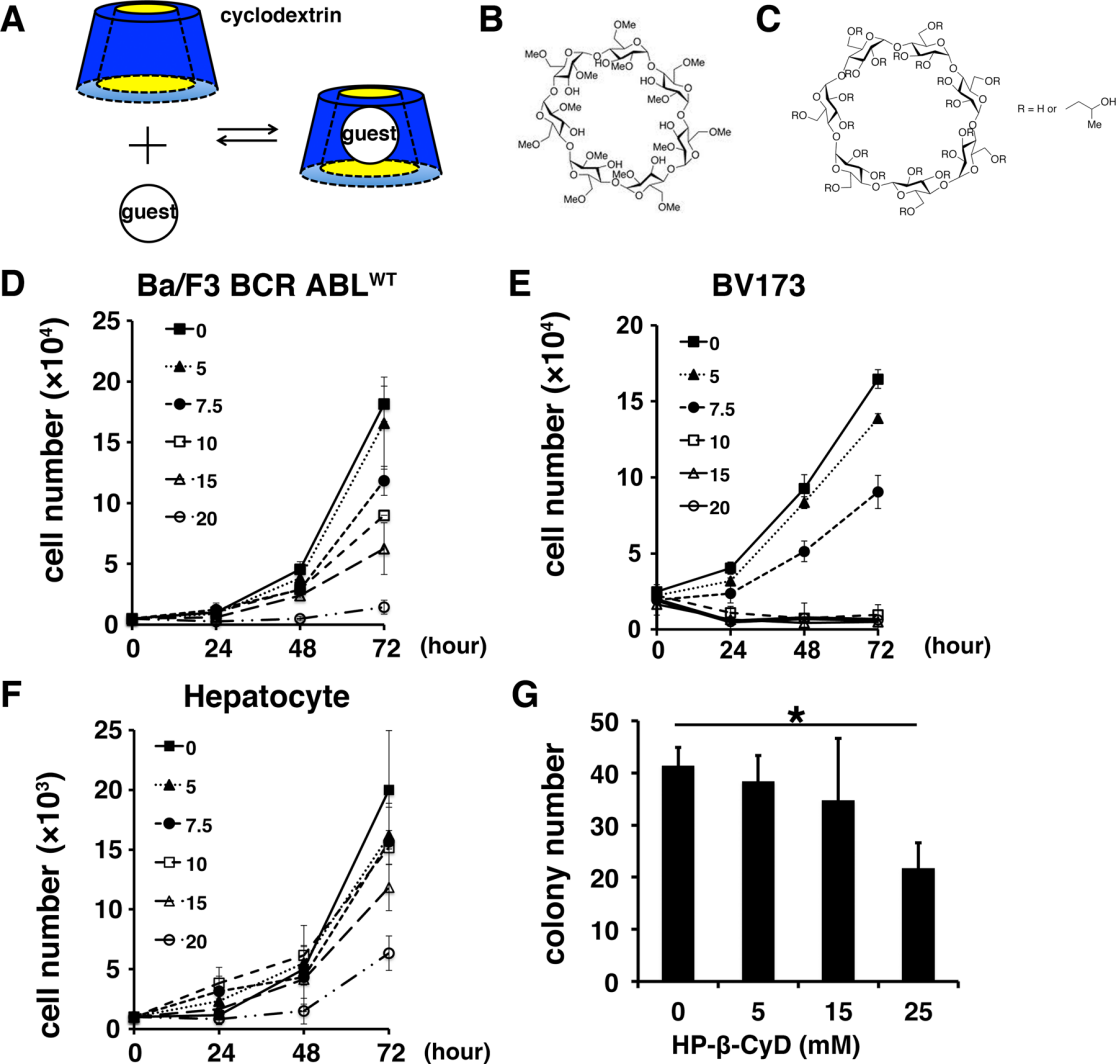
Function and structure of cyclodextrins, and effects of HP-β-CyD on leukemic cell growth. (**A**) Cyclodextrins (CyDs) can form water-soluble complexes with lipophilic guests through encapsulation into the cavity of CyDs. (**B** and **C**) Chemical structure of methyl-β-cyclodextrin (M-β-CyD) (**B**), and 2-hydroxypropyl-β-cyclodextrin (HP-β-CyD) (**C**). (**D–F**) Growth curves for each cell line were determined by trypan blue dye exclusion. Ba/F3 BCR-ABL^WT^ cells (**D**), BV173 cells (E), and hepatocytes (**F**) were exposed to 0 mM (■), 5 mM (▲), 7.5 mM (●), 10 mM (□), 15 mM (△), 20 mM (○) HP-β-CyD. Data are the mean ± SD of at least three independent experiments. (**G**) Colony-forming capacity of murine bone marrow mononuclear cells treated with HP-β-CyD. Data represent the mean number of colonies ± SD (n = 3). *P < 0.05.

Cyclodextrins (CyDs) are cyclic oligosaccharides, which have hydrophilic external faces and hydrophobic internal environments. The internal cavity of CyD has the ability to encapsulate lipophilic compounds and solubilize them in aqueous solutions (Fig 1A) [16]. CyDs are widely used in the pharmaceutical industry because of their ability to improve drug solubility and bioavailability [17,18]. CyDs interact with cell membrane components, such as cholesterol and phospholipids, resulting in the induction of hemolysis at high concentrations [19,20]. Amongst them, methyl-β-cyclodextrin (M-β-CyD) (Fig 1B) is used as a lipid raft disrupting agent through extraction of cholesterol and sphingolipids from these microdomains [21–24]. Several *in vitro* studies demonstrated that M-β-CyD induces apoptosis of various cancer cells [25–27]. However, systemic use of M-β-CyD is limited because of potential toxicities, such as its hemolytic effect on erythrocytes, and nephrotoxicity due to its high surface activity and inclusion ability [19,28,29].

2-Hydroxypropyl-β-cyclodextrin (HP-β-CyD) (Fig 1C) is used clinically as a pharmaceutical excipient for poorly water-soluble drugs. HP-β-CyD is available in registered oral, buccal, rectal, ophthalmic, and intravenous products [16]. Oral and intravenous solutions containing HP-β-CyD in complex with itraconazole, a broad-spectrum triazole antifungal agent, are widely used [30]. Furthermore, HP-β-CyD has recently been approved for the treatment of Niemann-Pick Type C disease (NPC), a lysosomal lipid storage disorder [31,32]. However, the potential anticancer activities of HP-β-CyD have not yet been noted. Given that cellular cholesterol accumulates in many malignancies and that HP-β-CyD is already used in clinical practice, we suspected that HP-β-CyD itself could be used as a novel anticancer agent by directly modulating cholesterol homeostasis in cancer cells. We therefore investigated anticancer activities of HP-β-CyD using various leukemic cell lines *in vitr*o and leukemia mouse models *in vivo*.

## Materials and Methods

### Reagents and cell lines

HP-β-CyD was purchased from Tokyo Chemical Industry (Tokyo, Japan). M-β-CyD was obtained from Junsei Chemical (Tokyo, Japan). The average molecular weight of HP-β-CyD and M-β-CyD are 1391 and 1302, respectively. For *in vitro* use, compounds were dissolved in saline (Otsuka Pharmaceuticals, Tokyo, Japan) to a concentration of 500 mM. For *in vivo* use, HP-β-CyD and M-β-CyD were dissolved in saline to a concentration of 150 mM and 50 mM, respectively. These were stored at 4°C until use.

The human leukemic cell lines, BV173, K562, NALM6, HL60, Jurkat, and MOLT4, were purchased from the American Type Culture Collection (ATCC, Manassas, VA, USA). KBM-5 and KBM-5/STI cell lines were kindly provided by Dr M. Beran (The University of Texas M.D. Anderson Cancer Center, Houston, TX, USA) [33]. An imatinib-sensitive cell line, MYL, and an imatinib-resistant Lyn-overexpressing cell line, MYL-R, were kindly provided by Dr H. Tanaka (Hiroshima City Asa Hospital, Hiroshima, Japan) [34]. Two hypoxia-adapted (HA)-CML cell lines, K562/HA and KCL22/HA were generated and cultured under 1.0% O_2_ as described previously [35]. Ba/F3 BCR-ABL^WT^ were generated as described previously [36]. Cells were maintained as suspension cultures in RPMI1640 (Sigma-Aldrich, St. Louis, USA) supplemented with 10% heat-inactivated fetal calf serum (FCS; Nichirei Bioscience Inc., Tokyo, Japan) at 37°C in a fully humidified chambers containing 5% CO_2_ in air. Studies with human samples were approved by the Ethical Board of Saga University Hospital. Bone marrow (BM) or peripheral blood (PB) samples were obtained with written informed consent according to the Declaration of Helsinki from newly diagnosed CML and AML patients.

### Mice

5- to 6-week-old Balb/cAJcl-*nu/nu* (nude) mice and 8-week-old NOD/ShiJic-*scid* Jcl (NOD/SCID) mice were purchased from CLEA Japan, Inc. (Osaka, Japan), and used for *in vivo* transplantation experiments. All animal experiments were performed in accordance with the institutional guidelines of Saga University and with the approval of the Saga University Institutional Review Board.

### Effects of HP-β-CyD on *in vitro* cell growth

Cell viability was assessed using a trypan blue dye exclusion method and cell proliferation was evaluated using a modified methyl-thiazol-diphenyl- tetrazolium (MTT) assay with SF reagent (Nacalai Tesque, Kyoto, Japan) as described previously [37,38]. Cells, including human primary hepatocytes (Applied Cell Biology Research Institute, Kirkland, WA, USA) were seeded in flat-bottomed 96-well plates (Greiner Labortechnik, Hamburg, Germany) at a density of 1×10^4^ cells in 100 μL medium per well, and incubated with HP-β-CyD at various concentrations for 72 hours. The mean of three replicates was calculated for each concentration. Half-maximal inhibitory concentration values (IC_50_) were determined by nonlinear regression using CalcuSyn software (Biosoft, Cambridge, UK).

### Western blot analysis

Whole cell lysates of leukemic cells treated with or without HP-β-CyD were prepared from cells using lysis buffer, as reported previously, with minor modification [39]. Protein was separated using a 10% NuPAGE electrophoresis system (Novex^®^, Life Technologies Co., Carlsbad, CA, USA), transferred to a nitrocellulose membrane (Schleicher & Schuell, BioScience GmbH, Dassel, Germany), blocked with 5% bovine serum albumin at room temperature for 1 hour, and incubated with primary antibodies at 4°C overnight. Antibodies against Akt, phosphorylated-Akt (Thr308 or Ser473), phospholyrated-Erk1/2 (Thr202/Thr204), phospholyrated-Stat5, Lyn (Cell Signaling Technology, Danvers, MA, USA), Stat5, Erk1/2, Actin (Santa Cruz Biotechnology, Santa Cruz, CA, USA), and phospholyrated-Lyn (Abcam, Cambridge, UK) were used as primary antibodies. Horseradish peroxidase-coupled immunoglobulin IgG (GE Healthcare, Buckinghamshire, UK) was used as the secondary antibody. An enhanced chemiluminescence kit (GE Healthcare) was used for detection. The results are representative of at least two independent experiments. Intensity of the immunoblot signals after background subtraction was quantified using ImageJ software.

### Cell-cycle analysis

Cell-cycle analyses of human leukemic cell lines were performed as described previously [39]. In brief, 1×10^6^ cells were treated with the indicated concentration of HP-β-CyD. Twelve or twenty-four hours after HP-β-CyD treatment, cells were collected and fixed with 70% ethanol. Cells were then incubated with 0.1% Triton X-100 and 0.5% RNase A at room temparature for 30 minutes and stained with 50 μg/mL propidium iodide (PI; BD Biosciences, San Jose, CA, USA). Cellular DNA content was analyzed by flow cytometry, and cell-cycle profiles were determined using a FACS Caliber flow cytometer with CellQuest software (BD Biosciences). Data are the mean ± SD of three independent experiments.

### Apoptosis assays

Apoptosis assay was performed by staining cells with 7-amino-actinomycin D (7-AAD) and annexin V (BD Biosciences), according to the manufacturer’s instructions. Cells were cultured in 6-well plate at a density of 4×10^5^ cells, and incubated with various concentrations of HP-β-CyD for 12 or 24 hours. Then, cells were stained with 7-amino-actinomycin D (7-AAD) and Annexin V-APC (BD Biosciences), and analyzed using a FACSAriaII system with Diva software (BD Biosciences). Data are the mean ± SD of three independent experiments.

### Hematopoietic colony-forming assays

HP-β-CyD toxicity in normal hematopoietic progenitors was investigated using a standard methylcellulose culture assay as described previously [40]. A total of 2×10^4^ mononuclear cells from the BM of 10-week-old C57BL/6N mice were exposed to 0, 5, 15, or 25 mM HP-β-CyD in 1 mL MethoCult M3434 (Stem Cell Technologies, Vancouver, Canada). After 8 days of culture, the number of colonies was counted using an inverted microscope. Data represent the mean number of colonies ± SD (n = 3).

Clinical samples were obtained with informed consent. Mononuclear cells from leukemia patients were cultured in semi-solid medium containing recombinant cytokines (MethoCult H4435; Stem Cell Technologies). Results are expressed as the percentage of colonies relative to the untreated control ± SD of three replicates.

### Cholesterol assays

Leukemic cells (3×10^6^) were incubated with 5 or 10 mM HP-β-CyD in HBSS (pH 7.4) at 37°C for 1, 2, or 3 hours. Cell culture supernatants were recovered by centrifugation (3,000 rpm, 5 min). The concentration of total cholesterol in the supernatants was determined using a Cholesterol E-test Wako (Wako Pure Chemical Industries, Osaka, Japan). Data are the mean ± SD of three experiments. Cellular lipids were extracted with methanol:chloroform (1:2), and total cholesterol and free cholesterol were determined enzymatically. The amount of esterified cholesterol was calculated by subtracting free cholesterol from total cholesterol. Cellular protein concentration was determined by BCA assay. Data are the mean ± SD of three experiments. For filipin staining, cells were incubated with β-CyDs (10 mM) for 1 hour. Thereafter, cellular cholesterol was detected using a Cholesterol Cell-Based Detection Assay Kit (Cayman Chemical, Ann Arbor, MI, USA).

### Murine leukemia model

Two different experimental settings were used. The protocol was approved by the Committee on the Ethics of Animal Experiments of the Saga University (Permit number: 25-028-0). First, nude mice were intravenously transplanted with 1×10^6^ EGFP^+^ Ba/F3 BCR-ABL^WT^ cells. These mice were intraperitoneally injected with 200 μL vehicle (saline, Otsuka Pharmaceuticals) or HP-β-CyD (50 or 150 mM) for 20 consecutive days 3 days after transplantation, and survival was monitored daily. Leukemic cell engraftment was confirmed by detection of GFP-positive cells in the recipient’s BM using flow cytometry.

The second experimental setting involved a human leukemia xenograft model. BV173 cells (1×10^6^) were intravenously injected into sublethally irradiated (2 Gy) NOD/SCID mice. After 72 hours, xenotransplanted mice were intraperitoneally injected with 200 μL vehicle or HP-β-CyD (50 or 150 mM) for 5 consecutive days every week for 13 weeks, and survival was monitored daily. The percentage of human leukemic cells in BM was determined by flow cytometry after double staining with FITC-conjugated anti-human CD19 (BD Biosciences) and PE/Cy7-conjugated anti-mouse CD45 (BioLegend, San Diego, CA, USA) antibodies. All surgery was performed under sodium pentobarbital anesthesia, and all efforts were made to minimize suffering. Mice were euthanized with ether when they became moribund or unable to obtain food or water, as recommended by the institutional guidelines of Saga University. Survival data were analyzed by a log-rank nonparametric test and shown as Kaplan-Meier survival curves (n = 10 for each group).

### Lung histology

HP-β-CyD was administered to NOD/SCID mice for 13 weeks as described in the above section entitled “Murine leukemia model”. Age-matched mice were used as a control. Lungs were perfused with 10% buffered formalin and excised. Tissues were fixed in 10% buffered formalin and embedded in paraffin. These blocks were then sectioned and stained with hematoxylin and eosin (H&E). Samples were reviewed by Dr C. Conti (Texas A&M Health Science Center). Representative images from at least two samples are shown.

### Statistical analysis

Results are expressed as the mean ± standard deviation (SD). Data were analyzed using an unpaired two-tailed Student’s *t*-test. Values of *P* <0.05 were considered significant. To analyze *in vivo* efficacy, survival curves were created using the Kaplan-Meier method and compared with a log-rank test.

## Results

### HP-β-CyD inhibits the growth of various leukemic cell lines

First, we evaluated the effect of HP-β-CyD on leukemic cell viability. HP-β-CyD significantly inhibited the growth of mouse Pro-B cell Ba/F3 cells expressing wild-type BCR-ABL (hereafter Ba/F3 BCR-ABL^WT^) (Fig 1D), and BV173 cells derived from a human Philadelphia chromosome-positive (Ph^+^) acute leukemia patient, in a dose- and time-dependent manner (Fig 1E). Notably, HP-β-CyD did not significantly interfere with growth of normal human primary hepatocytes at concentrations of up to 15 mM (Fig 1F).

IC_50_ values of HP-β-CyD were determined in 13 leukemia cell lines (Ph^+^ leukemia; BV173, K562, KBM-5, KBM-5/STI [33], KCL22, MYL and MYL-R [34], AML; HL-60, pre-B cell leukemia; NALM-6, T-cell leukemia; Jurkat and MOLT-4, adult T-cell leukemia; MT1, and MT2). The IC_50_ values for HP-β-CyD after 72 hours exposure were in the range of 3.86–10.09 mM (Table 1). Interestingly, the HP-β-CyD-induced cell growth inhibition of imatinib-resistant cell lines, such as KBM-5/STI and MYL-R, was equivalent to that of the respective parental cells. The IC_50_ value of HP-β-CyD in hepatocytes (18 mM) was approximately three-fold higher than those of the leukemic cell lines examined.

**Table 1:** IC_50_ values of HP-β-CyD in various leukemic cell lines.

	cell line	IC_50_ (mM)	SD
**Ph** ^**+**^ **leukemia**	**BV173**	**4.68**	**0.98**
	**K562**	**7.02**	**0.32**
	**KBM5**	**6.99**	**2.02**
	**KBM5/STI**	**8.47**	**0.6**
	**MYL**	**10.09**	**0.35**
	**MYL-R**	**7.34**	**1.8**
	**KCL22**	**8.06**	**0.71**
**Acute myeloid leukemia**	**HL60**	**8.26**	**1.03**
**Pre-B cell leukemia**	**NALM6**	**5.75**	**0.51**
**Mouse proB harboring BCR-ABL**	**Ba/F3 BCR-ABL** ^**WT**^	**6.01**	**1.04**
**ATL**	**MT1**	**8.23**	**1.17**
	**MT2**	**6.18**	**0.52**
**T-cell leukemia**	**Jurkat**	**4.73**	**0.61**
	**MOLT4**	**8.62**	**0.23**
**normal control**	**hepatocyte**	**18.65**	**4.84**

Values represent the mean ± SD of at least three independent experiments.

To determine the therapeutic window of HP-β-CyD, the susceptibility of normal hematopoietic progenitors to HP-β-CyD was examined using a colony formation assay. Under control conditions, 2×10^4^ C57BL/6N BM cells formed 41 ± 3.5 colonies after 8 days of culture. When cells were treated with 5, 15, or 25 mM HP-β-CyD, the percentage of colonies was 93 ± 8.6%, 84 ± 23.5%, and 52.4 ± 9.7% of control, respectively (Fig 1G). These results indicate that concentrations greater than 15 mM HP-β-CyD are required to suppress BM colony formation.

### HP-β-CyD inhibits cell growth by inducing apoptosis and G_2_/M cell-cycle arrest

**Fig 2:**
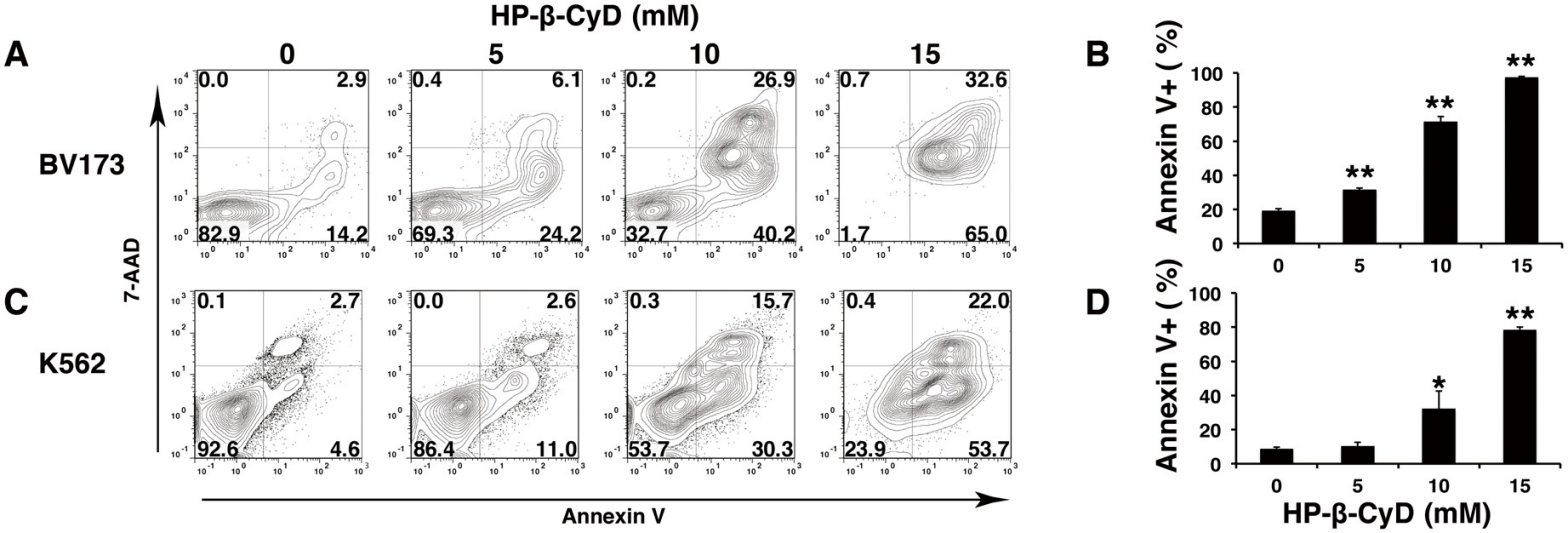
HP-β-CyD induces apoptosis in BV173 cells and K562 cells. (**A–D**) BV173 cells and K562 cells were treated with 0, 5 mM, 10 mM, 15 mM HP-β-CyD, respectively. After 24 hours of culture, cells were collected and stained with Annexin V and 7-AAD. (**A**) FACS plots, representative of three independent experiments using BV173 cells. (**B**) Percentage of Annexin V-positive BV173 cells after culture with HP-β-CyD for 24 hours. Data are the mean ± SD of three independent experiments. **P < 0.01. (**C**) FACS plots, representative of three independent experiments using K562 cells. (**D**) Percentage of Annexin V-positive K562 cells after culture with HP-β-CyD for 24 hours. Data are the mean ± SD of three independent experiments. *P < 0.05. **P < 0.01.

Cell apoptosis was determined by Annexin V and 7-AAD staining. HP-β-CyD increased apoptosis in both BV173 and K562 cells in a dose- and time-dependent manner (Fig 2).

**Fig 3:**
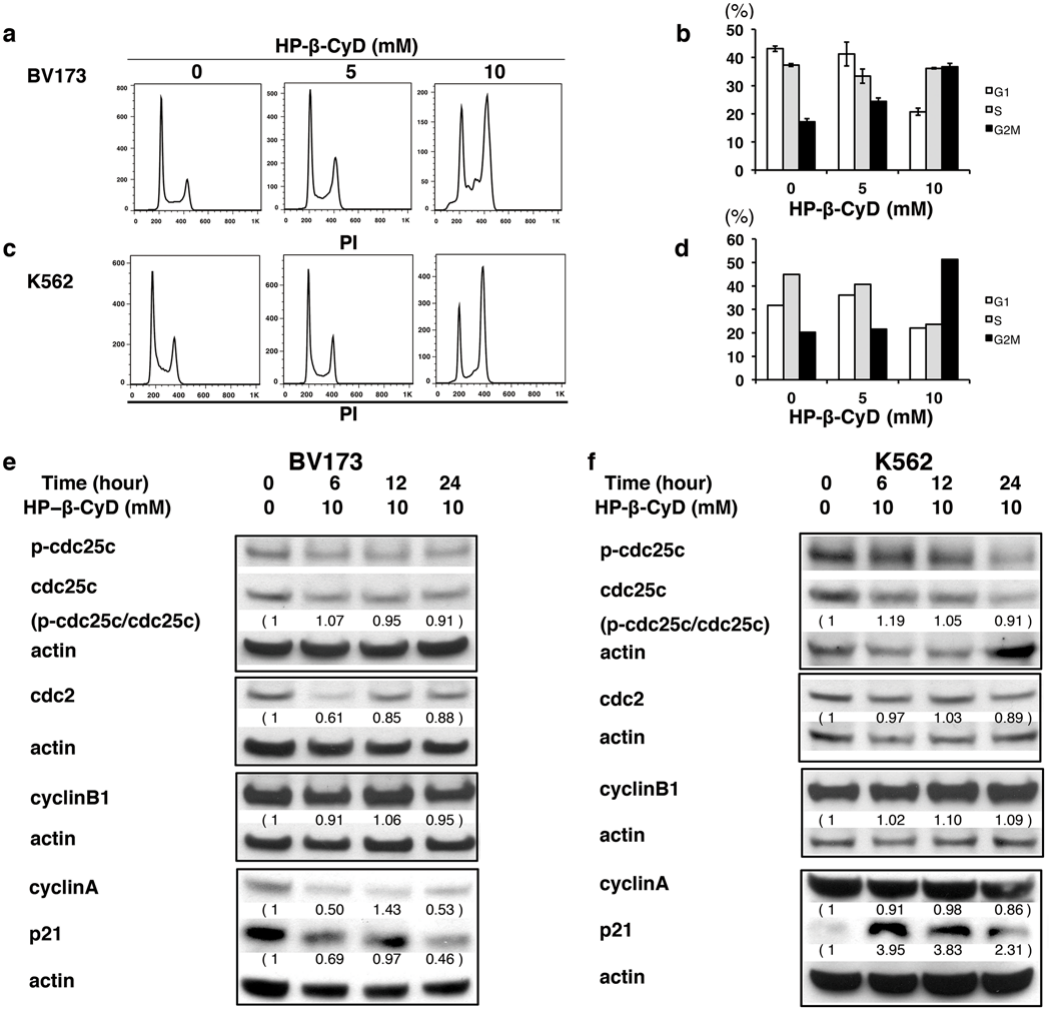
HP-β-CyD causes cell-cycle arrest in leukemic cells. (**A–D**) BV173 and K562 cells were treated with the indicated concentration of HP-β-CyD for 12 hours, then flow cytometric analysis of PI-stained nuclei was performed. (**A**) Representative flow cytometric histograms of PI-stained BV173 cells. (**B**) The percentage of cells in G_0_/G_1_, S, or G_2_/M phase was assessed in viable BV173 cells. White: G_1_-phase, gray: S-phase, black: G_2_/M-phase. (**C**) Representative flow cytometric histograms of PI-stained K562 cells. (**D**) The percentage of cells in G_0_/G_1_, S, or G_2_/M phase was assessed in viable K562 cells. White: G_1_-phase, gray: S-phase, black: G_2_/M-phase. (**E** and **F**) Effects of HP-β-CyD on the expression of G_2_/M cell-cycle-associated proteins. BV173 (**E**) and K562 cells (**F**) were treated with 10 mM HP-β-CyD for the indicated times. Cells were lysed and analyzed by Western blotting. Western blot images are representative results from at least two independent experiments. Detection of β-actin was used as a loading control. Intensity of the immunoblot signals after background subtraction was quantified using ImageJ software.

Furthermore, both BV173 (Fig 3A and 3B) and K562 cells (Fig 3C and 3D) responded to HP-β-CyD with a dose-dependent increase in the percentage of cells in G_2_/M phase. As the percentage of cells in G_2_/M increased, the percentage of G_1_ phase decreased, whereas the proportion of S-phase cells was not significantly altered by HP-β-CyD treatment. Treatment with HP-β-CyD also induced G_2_/M cell-cycle arrest in another Ph^+^ cell line, KBM5, and Ph-negative cell lines such as NALM-6, Jurkat, and MOLT4 (S1 Fig). Next, the effects of HP-β-CyD treatment on the expression of G_2_/M cell-cycle regulators, such as cdc25C, cdc2, cyclin A, cyclin B1, and p21, were examined by western blot analysis. HP-β-CyD had no effect on p-cdc25c and cyclin B1 protein expression; however, levels of cdc2 slightly decreased in BV173 cells (Fig 3E and 3F). Cyclin A and p21 decreased in BV173 cells, while in K562 cells, p21 expression was induced after 6 hours of HP-β-CyD treatment and slowly declined thereafter (Fig 3E and 3F). p21 is a cell cycle regulatory protein that can cause cell cycle arrest [41]. However, the role of p21 is complex because it also posseses pro- and anti-apoptotic abilities, and the function of p21 in apoptosis is cell type and cellular context specific [42–46]. Therefore, different behavior of p21 in HP-β-CyD-treated BV173 and K562 cells may be explained in part by the multiple roles of p21. These data indicate that HP-β-CyD-induced cell growth inhibition could be, in part, ascribed to induction of apoptosis and cell-cycle arrest at the G_2_/M checkpoint.

### HP-β-CyD disturbs leukemic cell cholesterol homeostasis

**Fig 4:**
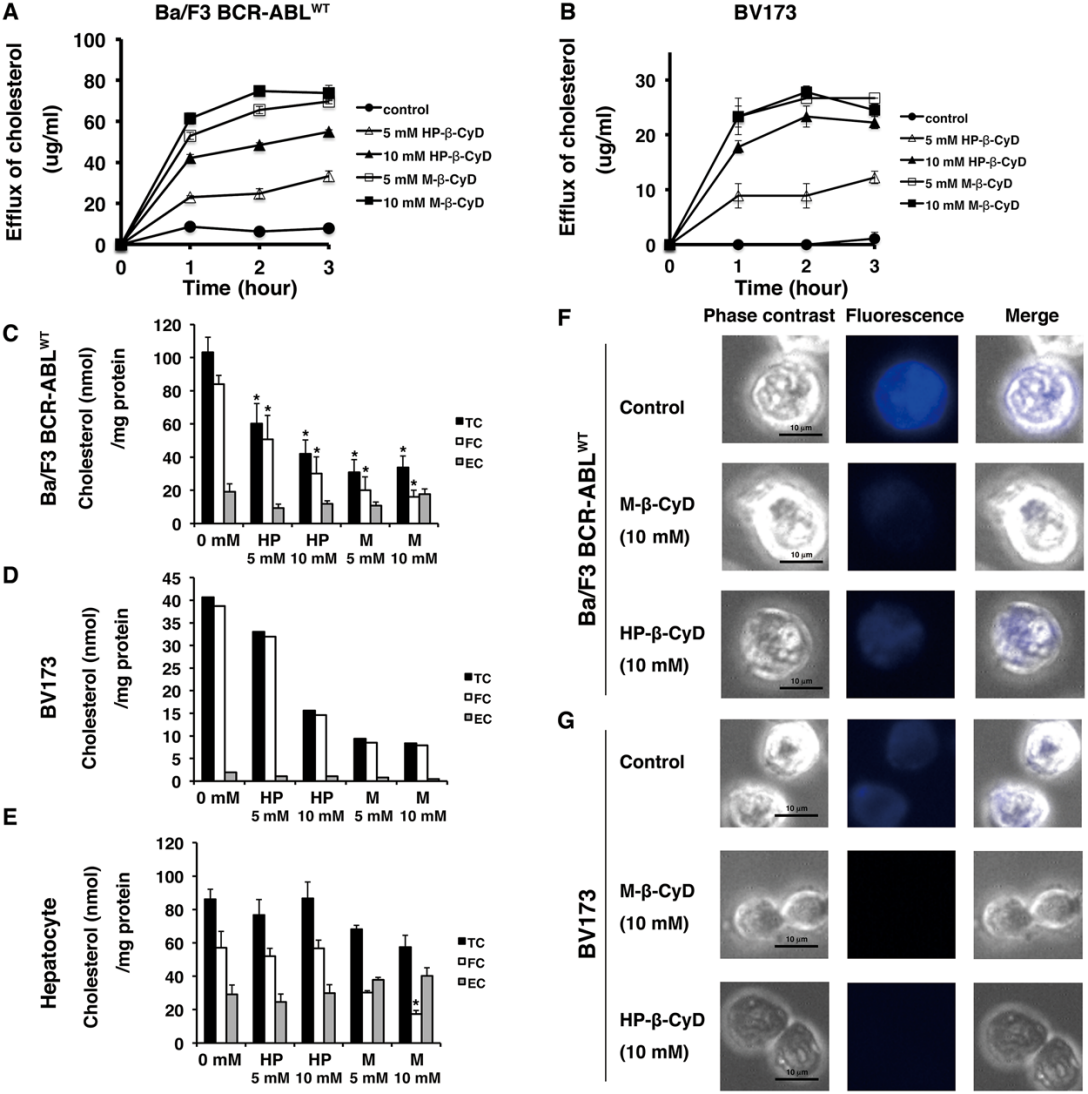
Disruption of cellular cholesterol homeostasis by β-CyDs. (**A** and **B**) Effect of β-CyDs on cholesterol efflux from Ba/F3 BCR-ABL^WT^ and BV173 cells. Ba/F3 BCR-ABL^WT^ cells (**A**) and BV173 cells (**B**) were incubated with β-CyDs (5 mM, 10 mM) for 1, 2, or 3 hours. Then the cholesterol concentration of the culture supernatants was determined. (●), control; (△), 5 mM HP-β-CyD; (▲), 10 mM HP-β-CyD; (□), 5 mM M-β-CyD; (■), 10 mM M-β-CyD. Data are the mean ± SD of three experiments. (**C**–**E**) Measurement of the intracellular cholesterol content of β-CyD-treated cells. Ba/F3 BCR-ABL^WT^ (**C**), BV173 (**D**), and hepatocytes (**E**) were incubated with vehicle, HP-β-CyD (5 mM, 10 mM), or M-β-CyD (5 mM, 10 mM), respectively. After 1 hour, cellular lipids were extracted, and cholesterol contents were determined. HP, HP-β-CyD; M, M-β-CyD; TC, total cholesterol; FC, free cholesterol; EC, esterified cholesterol. (**F** and **G**) Images of filipin staining for Ba/F3 BCR-ABL^WT^ (**F**) or BV173 cells (**G**) treated with 10 mM β-CyDs for 1 hour are shown. Scale bar: 10 μm.

We recently reported that M-β-CyD induces apoptosis in some solid cancer cell lines, possibly due to cholesterol depletion from cell membranes [27]. Therefore, to clarify whether HP-β-CyD-induced apoptosis is also associated with cholesterol depletion, we evaluated the effect of CyDs on cholesterol efflux from leukemic cells. HP-β-CyD or M-β-CyD treatment both increased cholesterol release in a time- and dose-dependent manner. Greater concentrations of cholesterol were released from Ba/F3 BCR-ABL^WT^ cells treated with M-β-CyD than from cells treated with HP-β-CyD (Fig 4A). Similar results were also observed in BV173 cells and human hepatocytes (Fig 4B and S2A Fig). Next, we determined intracellular concentrations of cholesterol in leukemic cells treated with β-CyDs. Treatment with β-CyDs decreased cholesterol content in a dose-dependent manner (Fig 4C and 4D). Contrary to the results of the cholesterol efflux experiment, the cholesterol content of cells incubated with M-β-CyD was lower than that of the HP-β-CyD-treated cells (Fig 4C and 4D). Surprisingly, hepatocyte cholesterol content was not affected by treatment with the same concentration of HP-β-CyD (Fig 4E).

The reduced intracellular cholesterol content of CyDs-treated cells was also confirmed by filipin staining, which is a polyene antibiotic that specifically binds to cholesterol [47]. Although control (untreated) cells were positive for filipin, both M-β-CyD and HP-β-CyD-treated leukemic cells exhibited lower levels of filipin labeling than control cells (Fig 4F and 4G). In particular, M-β-CyD-treated cells demonstrated negligible labeling of filipin (Fig 4F and 4G). By contrast, hepatocytes treated with HP-β-CyD showed similar levels of filipin labeling to control cells (S2B Fig). These data support the results of the cholesterol efflux and intracellular cholesterol assays. The ability of M-β-CyD to markedly reduce cholesterol levels may reflect the degree of its cellular toxicity.

### Effect of HP-β-CyD on signal transduction pathways involved in leukemia cell proliferation and survival

**Fig 5:**
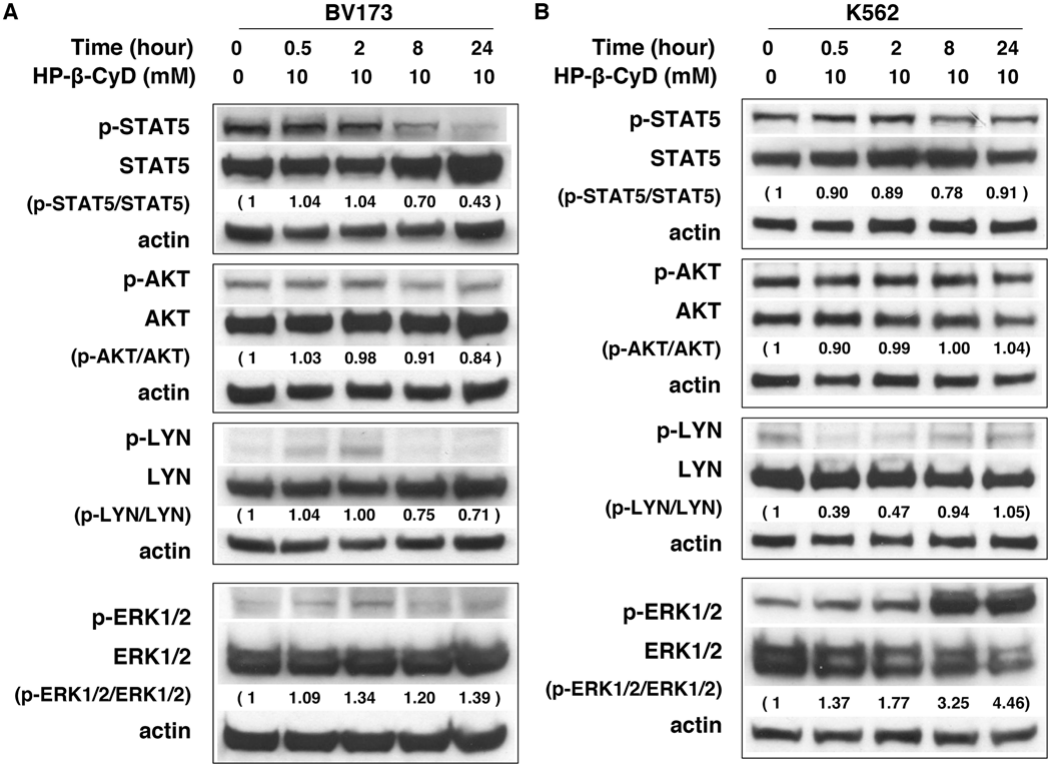
Protein expression and phosphorylation of BCR-ABL-associated kinases in leukemic cell lines treated with HP-β-CyD. BV173 and K562 cells were treated with 10 mM HP-β-CyD for the indicated times, after which STAT5, AKT, LYN, and ERK1/2 protein levels, and the phosphorylation status of each protein were determined by western blot analysis. Detection of β-actin was used as a loading control. Data are representative of three independent experiments. Intensity of the immunoblot signals after background subtraction was quantified using ImageJ software, and the relative intensities of p-STAT5, p-AKT, p-LYN, and p-ERK1/2 compared to those of STAT5, AKT, LYN, and ERK1/2 were respectively calculated.

Signal transduction pathways such as RAS/RAF/MAPK, PI3K-AKT, STAT5, and Lyn, are activated in leukemia cells [48–50]. Thus, we next conducted experiments to elucidate the molecular mechanism by which HP-β-CyD inhibits the growth of leukemic cells. BV173 and K562 cells were exposed to 10 mM HP-β-CyD for varying times up to 24 hours, then changes in the expression and phosphorylation status of Akt, Erk, Stat5, and Lyn were examined. In BV173 cells, HP-β-CyD treatment markedly inhibited Stat5 phosphorylation. Akt and Lyn were also dephosphorylated on the treatment with HP-β-CyD, whereas the levels of phosphorylated (p)-ERK1/2 slightly increased in a time-dependent manner (Fig 5A). In K562 cells, HP-β-CyD treatment reduced levels of p-Lyn, which is known to reside in lipid rafts [51] after 0.5 and 2 hours of treatment, but p-Lyn recovered after 8 and 24 hours of treatment. The levels of p-ERK1/2 increased, with no significant effect on p-Stat5 or p-Akt observed (Fig 5B). Though BV173 and K562 cell lines were established from patients with Ph^+^ chronic myeloid leukemia in blast crisis [52,53], these two cell lines have some different characteristics [53,54]. Concordant with reports concerning M-β-CyD [10,25,26], these results suggest that HP-β-CyD effects on signal transduction may be cell context dependent.

### HP-β-CyD inhibits the proliferation of a tyrosine kinase inhibitor (TKI)-resistant cell line

Although first- and second-generation TKIs, such as imatinib, nilotinib, and dasatinib, demonstrate improved outcomes in patients with CML and Ph^+^ acute lymphoblastic leukemia, a subset of patients with BCR-ABL mutations, particularly the T315I mutation, exhibit resistance to these TKIs [3]. Currently, the third-generation TKI, ponatinib, is the only TKI that demonstrates notable activity against the T315I mutation [55].

In light of the data showing the inhibitory effect of HP-β-CyD on the proliferation of CML cell lines, we were prompted to evaluate the potential of HP-β-CyD to overcome TKI resistance. The results of a modified MTT assay using TKIs and HP-β-CyD are shown in Table 2. Ba/F3 cells expressing the T315I mutant (Ba/F3 BCR-ABL^T315I^) were resistant to imatinib and dasatinib [56]. By contrast, Ba/F3 BCR-ABL^T315I^ cell growth was inhibited by ponatinib (IC_50_: 6.3 nM). These results are consistent with previous reports [55,56]. Most interestingly, the IC_50_ of HP-β-CyD against Ba/F3 BCR-ABL^T315I^ cells was comparable to that of the Ba/F3 BCR-ABL^WT^ cells. Similar results were obtained in cell proliferation assays that used trypan blue staining (Fig 1D and S2 Fig). These results suggest that HP-β-CyD can inhibit the proliferation of TKI-resistant cell lines and may be of use against TKI resistance.

**Table 2:** Comparison of the IC_50_ values of TKIs and HP-β-CyD in Ba/F3 BCR-ABL^WT^ and Ba/F3 BCR-ABL^T315I^ cells.

		IC_50_		
cell line	Imatinib, μM	Dasatinib, nM	Ponatinib, nM	HP-β-CyD, mM
**BaF3/BCR-ABL** ^**WT**^	**0.09 ± 0.09**	**0.58 ± 0.15**	**0.89 ± 0.06**	**6.01 ± 1.04**
**BaF3/BCR-ABL** ^**T315I**^	**8.90 ± 1.21**	**2755.09 ± 120.15**	**6.30 ± 0.26**	**6.87 ± 0.76**
**ratio (T315I/WT)**	**102.85**	**4727.69**	**7.06**	**1.14**

Values shown are the mean ± SD of at least three independent experiments.

### Administration of HP-β-CyD prolongs survival in leukemia mouse models

**Fig 6:**
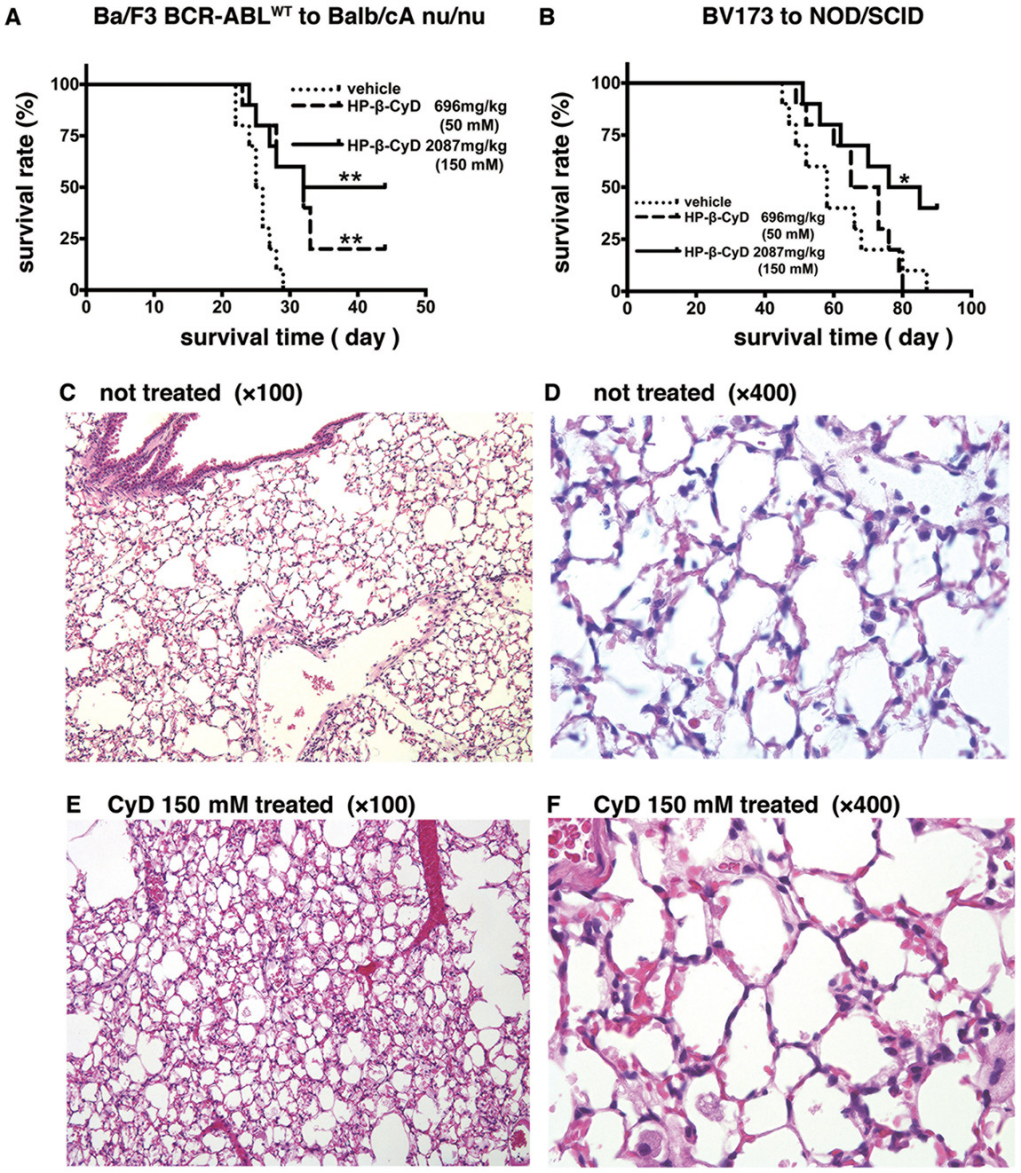
Effect of HP-β-CyD on survival in leukemia mouse models. (**A**) Survival of mice that received EGFP^+^ Ba/F3 BCR-ABL^WT^ cells. EGFP^+^ Ba/F3 BCR-ABL^WT^ cells (1×10^6^) were transplanted into 6-week-old nude mice. Three days after transplantation, 200 μL vehicle, 50 mM (695.5 mg/kg) HP-β-CyD, or 150 mM (2086.5 mg/kg) HP-β-CyD were intraperitoneally injected twice a day for 20 days, and survival was monitored daily. Gray lines, dotted lines, and black lines indicate the survival curves of vehicle-, 50 mM HP-β-CyD- and 150 mM HP-β-CyD-treated mice, respectively. Survival data were analyzed using a log-rank nonparametric test and are shown as Kaplan-Meier survival curves (n = 10). **P < 0.01. (**B**) Survival of human leukemia xenografted mice transplanted with BV173 cells. BV173 cells (1×10^6^) were intravenously injected into 2 Gy irradiated NOD/SCID mice. Three days after transplantation, 200 μL vehicle, 50 mM HP-β-CyD (695.5 mg/kg HP-β-CyD), or 150 mM HP-β-CyD (2086.5 mg/kg HP-β-CyD) were administered intraperitoneally for 5 consecutive days every week for 13 weeks, and survival was monitored daily. Gray lines, dotted lines and black lines indicate the survival curves of vehicle-, 50 mM HP-β-CyD-, and 150 mM HP-β-CyD-treated mice, respectively. Survival data were analyzed using a log-rank nonparametric test and are shown as Kaplan-Meier survival curves (n = 10). *P < 0.05. (**C–F**) Histological examination of lungs from age-matched controls, and HP-β-CyD-treated NOD/SCID mice. (**C** and **D**) hematoxylin and eosin (H&E) stained control lung. (**C**) Original magnification, ×100; (**D**), ×400. (**E** and **F**) H&E stained 150 mM HP-β-CyD-treated lung. (**E**) Original magnification, ×100; (**F**), ×400. Representative images from at least two samples are shown.

The *in vitro* data presented above demonstrate that HP-β-CyD has a significant inhibitory effects on leukemic cell growth. Next, to investigate the *in vivo* efficacy of HP-β-CyD, we generated mouse models of BCR-ABL-induced leukemia, and administered HP-β-CyD. Two different experimental settings were used. First, nude mice transplanted with Ba/F3 BCR-ABL^WT^ were treated with 200 μL of vehicle, 50 mM HP-β-CyD [695.5 mg/kg intraperitoneal (i.p.); b.i.d] or 150 mM HP-β-CyD (2086.5 mg/kg i.p.; b.i.d) for 20 days beginning 3 days after transplantation. Ba/F3 BCR-ABL^WT^ cell engraftment was confirmed by flow cytometric detection of EGFP^+^ cells in the BM of control mice (S4A Fig). All vehicle-treated mice died within 28 days. HP-β-CyD-treated mice survived longer than vehicle-injected mice, and the overall survival of recipients that received 50 mM or 150 mM HP-β-CyD was significantly higher than that of vehicle-injected mice (Fig 6A). Surprisingly, the survival time of HP-β-CyD-injected mice was longer than that of imatinib-treated mice in the same experimental setting, as performed in a previous study in our laboratory [57].

The second setting utilized a human leukemia xenograft model. BV173 cells were intravenously transplanted into sublethally irradiated NOD/SCID mice. Intraperitoneal injections of 50 mM HP-β-CyD, 150 mM HP-β-CyD, or vehicle were performed for 5 consecutive days every week for 13 weeks, beginning 3 days after transplantation. BV173 cell engraftment into BM was confirmed by flow cytometry (S4B Fig). HP-β-CyD-treated mice survived longer than control mice, and the log-rank test for overall survival showed statistically significant differences between vehicle- and 150 mM HP-β-CyD-treated recipients (Fig 6B).

It has been reported that HP-β-CyD shows only limited toxicity, although toxicity profiles of CyDs may differ according to the route of administration [58]. HP-β-CyD-treated mice exhibited no gross lesions upon macroscopic examination. No hemolysis or anemia was observed (S1 and S2 Tables). More recently, HP-β-CyD-induced lung toxicity has been paid attention in the treatment of patients with NPC disease [59,60]. However, no obvious changes in lung histology were observed in HP-β-CyD-treated mice (Fig 6C–6F). Notably, all 150 mM M-β-CyD-injected mice died of diffuse alveolar hemorrhage within 24 hours of intraperitoneal administration (data not shown). These results clearly demonstrate that *in vivo* administration of HP-β-CyD inhibits leukemic cell growth, resulting in improved survival times. Furthermore, HP-β-CyD was well tolerated in mice with low toxicity.

### HP-β-CyD inhibits proliferation of hypoxia-adapted leukemic cells and human primary leukemic cell colony formation

We previously established two leukemic cell lines that survive and proliferate under 1.0% O_2_ hypoxic conditions [35]. These hypoxia-adapted (HA)-leukemic cells, namely, K562/HA and KCL22/HA, exhibit properties similar to leukemic stem cells, including relative cell dormancy, increase of side population fraction, resistance to Abl TKIs, and good engraftment ability. To investigate the effect of HP-β-CyD on leukemic stem-like cells, K562/HA and KCL22/HA cells were treated with HP-β-CyD. The IC_50_ values of HP-β-CyD in K562/HA and KCL22/HA cells were comparable to that of their parental cells (Table 3), suggesting that HP-β-CyD targets not only proliferating leukemia cells but also dormant leukemic stem cells. Apoptosis and G_2_/M cell-cycle arrest were also induced in K562/HA and KCL22/HA cells by HP-β-CyD treatment (S5 Fig).

**Table 3:** IC_50_ values of HP-β-CyD in hypoxia-adapted leukemic cell lines.

	cell line	IC_50_ (mM)	SD
**Ph** ^**+**^ **leukemia**	**K562**	**7.02**	**0.32**
	**K562/HA**	**3.86**	**0.85**
	**KCL22**	**8.06**	**0.71**
	**KCL22/HA**	**5.61**	**0.3**
**Normal control**	**hepatocyte**	**18.65**	**4.84**

Values represent the mean ± SD of at least three independent experiments.

**Fig 7:**
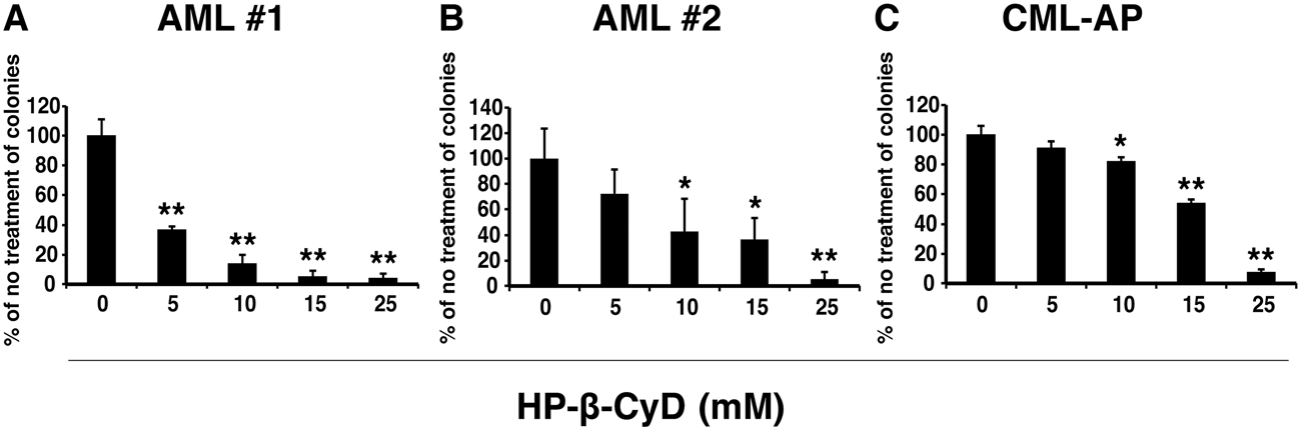
HP-β-CyD inhibits the in vitro colony-forming ability of human primary leukemic cells. Mononuclear cells (MNCs) obtained from two patients with AML and from one patient with accelerated phase CML (CML-AP) were plated in methylcellulose containing HP-β-CyD and cultured for 8–13 days. Colonies (>50 cells) were counted. Results are expressed as the percentage of colonies relative to the untreated control ± SD of three replicates. (**A** and **B**) Colony formation assays of MNCs from AML patients (FAB: M0) in the presence of HP-β-CyD. (**C**) Colony formation assays of MNCs from the CML-AP patient in the presence of HP-β-CyD. *P < 0.05. **P < 0.01.

We also evaluated the efficacy of HP-β-CyD in colony formation assays using mononuclear cells from two AML patients and from one accelerated phase CML patient. HP-β-CyD inhibited the formation of colonies in a concentration-dependent manner regardless of disease differences (Fig 7).

## Discussion

In the present study, we provide evidence that HP-β-CyD-mediated cholesterol efflux inhibits leukemic cell proliferation. M-β-CyD and HP-β-CyD have been widely used as cholesterol-depletion agents. We previously showed that M-β-CyD had potent cytotoxic activity, and the involvement of cholesterol depletion in cell-death by M-β-CyD [27]. We and others demonstrated that M-β-CyD have higher cholesterol extraction ability than HP-β-CyD, probably because of the increase in hydrophobic space of the CyD cavity [61–63]. Thus, M-β-CyD is highly toxic. Yan et al. reported that M-β-CyD induces programmed cell death in CML cells and has anti-leukemic effects when used in combination with imatinib against imatinib-resistant CML cells *in vitro* [26]. Our data revealed that single use of HP-β-CyD effectively inhibits not only the growth of various leukemia cells, such as AML, ALL and CML, but also imatinib-resistant CML cell growth, including cells harboring the T315I mutation (which are resistant to first- and second-generation TKIs), and stem cell-like CML cells, indicating that HP-β-CyD has an anti-leukemic potential irrespective of leukemia type or the different characteristics. *In vivo*, systemic administration of HP-β-CyD in murine leukemia models further demonstrated that HP-β-CyD suppresses leukemia progression, resulting in improved survival. These results suggest that HP-β-CyD is a potential novel anticancer agent.

Rapidly proliferating cells may conceivably require cholesterol for new membrane synthesis. Hence, increased endogenous cholesterol biosynthesis could be a common property of cancer. Statins, inhibitors of HMG-CoA reductase, exert growth inhibitory responses in leukemia cells *in vitro* [7,64,65]. These promising *in vitro* data led researchers to investigate possible clinical applications of statins as a single chemotherapeutic agent. Although conflicting results have been reported regarding their efficacy against cancers, including leukemia [66,67], the discrepancy between preclinical and clinical data may be explained in part by the difference in statin concentrations used. To achieve plasma statin concentrations comparable to those that demonstrated *in vitro* anticancer effects, considerably higher doses that may cause adverse effects, such as myopathy, rhabdomyolysis, and hepatotoxicity, must be administered. Recently, a leukemia-stroma co-culture system was used to screen for inhibition of stem cell properties, and identified 155 small-molecule compounds, including an HMGCR inhibitor, lovastatin, which preferentially inhibited leukemia stem cells (LSC) *in vitro* while sparing normal hematopoietic stem and progenitor cells. However, *in vivo* anti-LSC effect of lovastatin remains to be determined [68].

HP-β-CyD induced apoptosis and G_2_/M cell-cycle arrest *in vitro*, and prolonged survival (without severe toxicity) in murine leukemia models. The mechanisms by which HP-β-CyD induced apoptosis of cancer cells remain unclear. Rosenbaum et al. showed that M-β-CyD/dextran conjugates enter Niemann-Pick Type C1 (NPC1)-defective cells and localize to cholesterol-enriched late endosomes/lysosomes [69]. Plazzo et al. also demonstrated that fluorescein-tagged M-β-CyD is internalized into HeLa cells via clathrin-dependent endocytosis [70]. However, Onodera et al. demonstrated that although tetramethylrhodamine isothiocyanate (TRITC)-M-β-CyD is associated with apoptosis of various cancer cells, TRITC-M-β-CyD is not internalized, suggesting that M-β-CyD may be membrane impermeable [27]. These contradictive results could be caused by different concentrations of M-β-CyD or different cell types; thus, further studies are required to better understand how HP-β-CyD induces cancer cell apoptosis.

Accumulating evidence indicates that many different signaling proteins are affected by CyDs. For example, M-β-CyD treatment decreases Akt phosphorylation in a time-dependent manner in human epidermoid carcinoma cells [10], whereas M-β-CyD has no effect on the phosphorylation of Akt, PLCγ1, and ERK1/2 in human megakaryoblastic leukemia cells [71]. Yan et al. reported that treatment of K562 cells with M-β-CyD resulted in a significant decrease in ERK1/2 phosphorylation, and thus concluded that an ERK/SPK1 signaling pathway may be an important mediator of M-β-CyD effects [26]. However, in our system, we observed that HP-β-CyD treatment increased p-ERK1/2 in a time-dependent manner with decreased p-Lyn levels. These phenomena could be explained by the observation that Lyn negatively regulates ERK activation [72]. In erythroleukemic cell line, K562 cells, the levels of p-Lyn recovered after 8 and 24 hours of HP-β-CyD treatment. Because Lyn plays an essential role for erythropoiesis and erythroleukemia development, HP-β-CyD-treated K562 cells might restored the levels of p-Lyn to survive [73,74]. More detailed biochemical analyses are required to characterize the exact molecular mechanisms modulated by HP-β-CyD in leukemia cells.

In this study, the concentration of HP-β-CyD required to induce cell death *in vivo* is greater than 2g/kg in BV173-xenografted leukemic mice. Matsuo et al. showed the effectiveness of intravenous HP-β-CyD treatment in two patients with Niemann-Pick type C disease (NPC) [31]. One patient was treated with approximately 2,500 mg/kg of HP-β-CyD (40,000mg, intravenously administered for 8h), a concentration comparable to our *in vivo* experiment (Fig 6 150 mM HP-β-CyD). Tanaka et al measured serum HP-β-CyD concentrations in this patient [75]. Serum HP-β-CyD concentrations were 1,295, 1,756 (1.3 mM), and 90 μg/mL at 2, 8, 12h after the start of infusion. Because HP-β-CyD was administered slowly (8h) in this patient, higher serum HP-β-CyD concentration would be obtained when the dripping speed is increased. Thus, the dose of HP-β-CyD used in the present study would be promising dose in a future clinical setting.

HP-β-CyD toxicity was also extensively investigated in mice in our study. HP-β-CyD is well tolerated in most species and shows limited toxicity, depending on dose and route of administration [58]. A study using acute intraperitoneal HP-β-CyD administration revealed that, in mice, up to 10,000 mg/kg HP-β-CyD, is tolerable [76]. Although CyD-induced hemolysis of human and rabbit red blood cells is well known [19,20], in the present study, neither single nor repeated HP-β-CyD administration resulted in hemolysis or anemia in mice. Furthermore, HP-β-CyD-induced lung toxicity, such as foamy macrophage infiltration, alveolitis, and pulmonary edema has been reported in several animal species [58,60]. The lungs of mice treated with HP-β-CyD for 13 weeks did not exhibit any histologic changes compared with those of control mice. No severe adverse effects were reported in two patients with NPC who received infusions of HP-β-CyD over 2 years [31]. Future studies are required to clarify the potential side effects of HP-β-CyD.

In conclusion, we have demonstrated that HP-β-CyD disrupts cholesterol homeostasis and inhibits the proliferation of leukemic cells by induction of apoptosis and cell-cycle arrest. Systemic HP-β-CyD administration has limited toxicity where examined. HP-β-CyD is also effective against mutated TKI-resistant clones and hypoxia-adapted cells, suggesting that HP-β-CyD has a broad anticancer effect irrespective of the cellular characteristics by modulating cholesterol homeostasis. To our knowledge, this paper is the first report to describe the efficacy of HP-β-CyD in leukemia *in vitro* and *in vivo*. Early-phase clinical trials are planned to verify the efficacy and safety of HP-β-CyD for treatment of leukemia.

## Supporting Information

S1 Fig HP-β-CyD induces cell-cycle arrest in leukemic cells. (**A**–**D**) Leukemic cells were treated with the indicated concentration of HP-β-CyD for 12 hours, then flow cytometric analysis of PI-stained nuclei was performed. The percentage of cells in G_0_/G_1_, S, or G_2_/M phase was assessed in viable leukemic cells. White: G_1_-phase, gray: S-phase, black: G_2_/M-phase. (**A**) NALM-6, (**B**) KBM5, (**C**) Jurkat, (**D**) MOLT-4 cells. Data are the mean ± SD of three independent experiments.
(PPTX)

S2 Fig Effects of β-CyDs on efflux of cholesterol from hepatocytes. (**A**) Hepatocytes (1 × 10^7^ cells) were incubated in HBSS (pH 7.4) with or without HP-β-CyD or M-β-CyD (0, 5, 10 mM) for 1 hour. The concentration of cholesterol in HBSS were determined by Cholesterol E-test Wako^®^. (**B**) Image of filipin staining for hepatocytes. Primary hepatocytes were incubated with M-β-CyD (10 mM) or HP-β-CyD (10 mM) for 1 hour. Then, cells were treated with Filipin solution, and were scanned with a fluorescence microscope.
(TIF)

S3 Fig Effect of HP-β-CyD on the growth of Ba/F3 BCR-ABL^T315I^ cells. Ba/F3 BCR-ABL^T315I^ cells were exposed to 0 mM (■), 5 mM (▲), 7.5 mM (●), 10 mM (□), 15 mM (△), and 20 mM (○) HP-β-CyD. Viable cells were counted by a trypan blue dye exclusion method. Data are the mean ± SD of three independent experiments.
(TIF)

S4 Fig Leukemic cell engraftment into bone marrow in the BCR-ABL-induced leukemic mouse models. (**A**) Flow cytometric histogram of EGFP-positive BM cells from untreated nude mice that received EGFP^+^ Ba/F3 BCR-ABL^WT^ cells. (**B**) Representative FACS plot of BV173 cell-transplanted NOD/SCID mice. BM cells of NOD/SCID mice were analyzed by FACS 4 weeks after BV173 cell transplantation using an anti-human CD19 antibody and anti-mouse CD45 antibody.
(TIF)

S5 Fig HP-β-CyD inhibits hypoxia-adapted cell growth by inducing apoptosis and G_2_/M cell-cycle arrest. (**A-B**) K562/HA cells and KCL22/HA cells were treated with 0, 5 mM, 10 mM, 15 mM HP-β-CyD, respectively. After 24 hours of culture, cells were collected and stained with Annexin V and 7-AAD. (**A**) Percentage of Annexin V-positive K562/HA cells after culture with HP-β-CyD for 24 hours. Data are the mean ± SD of three independent experiments. (**B**) Percentage of Annexin V-positive KCL22 cells after culture with HP-β-CyD for 24 hours. Data are the mean ± SD of three independent experiments. **P < 0.01. (**C**-**D**) HP-β-CyD causes cell-cycle arrest in hypoxia-adapted leukemic cells. K562/HA and KCL22/HA cells were treated with the indicated concentration of HP-β-CyD for 12 hours, then flow cytometric analysis of PI-stained nuclei was performed. (**C**) The percentage of cells in G_0_/G_1_, S, or G_2_/M phase was assessed in viable K562/HA cells. White: G_1_-phase, gray: S-phase, black: G_2_/M-phase. (**D**) The percentage of cells in G_0_/G_1_, S, or G_2_/M phase was assessed in viable KCL22/HA cells. White: G_1_-phase, gray: S-phase, black: G_2_/M-phase. Data are the mean ± SD of three independent experiments.
(PPTX)

S1 Table Red blood cell count in HP-β-CyD-injected nude mice. Data from CBC counts of peripheral blood collected by retro-orbital bleeding of vehicle-, and HP-β-CyD-injected nude mice. Data are mean ± SD of three mice.
(DOCX)

S2 Table Red blood cell count in HP-β-CyD-injected NOD/SCID mice. Data from CBC counts of peripheral blood collected by retro-orbital bleeding of vehicle-injected, and NOD/SCID mice that received 50 mM HP-β-CyD administration for 7 weeks. Data are average of two mice.
(DOCX)
