# Supplementary material for: 2-Hydroxypropyl-β-Cyclodextrin Acts as a Novel Anticancer Agent
Source: PLoS One. 2015 Nov 4;10(11):e0141946. doi: 10.1371/journal.pone.0141946 (PMC4633159; doi:10.1371/journal.pone.0141946)
Supplement: S1 Fig — (A–D) Leukemic cells were treated with the indicated concentration of HP-β-CyD for 12 hours, then flow cytometric analysis of PI-stained nuclei was performed. The percentage of cells in G0/G1, S, or G2/M phase was assessed in viable leukemic cells. White: G1-phase, gray: S-phase, black: G2/M-phase. (A) NALM-6, (B) KBM5, (C) Jurkat, (D) MOLT-4 cells. Data are the mean ± SD of three independent experiments. (PPTX) [file pone.0141946.s001.pptx]

## Slide 1
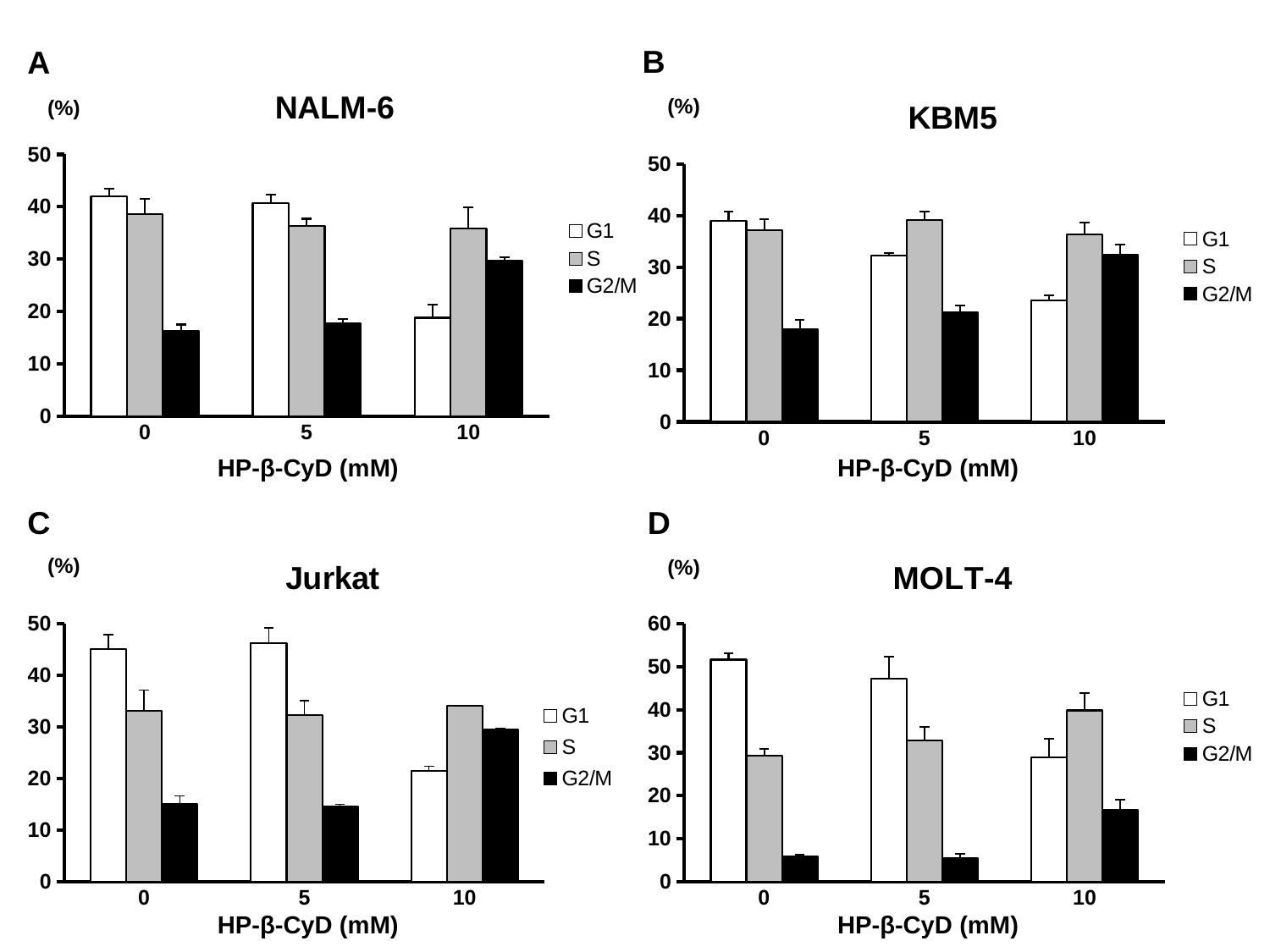

B
A
### Chart: NALM-6
| Category | G1 | S | G2/M |
|---|---|---|---|
| 0 | 41.93333333333334 | 38.56666666666667 | 16.3 |
| 5 | 40.733333333333334 | 36.4 | 17.73333333333333 |
| 10 | 18.8 | 35.9 | 29.666666666666668 |
### Chart: KBM5
| Category | G1 | S | G2/M |
|---|---|---|---|
| 0 | 39.03333333333333 | 37.13333333333333 | 17.999999999999996 |
| 5 | 32.199999999999996 | 39.13333333333333 | 21.233333333333334 |
| 10 | 23.600000000000005 | 36.4 | 32.4 |(%)
(%)
HP-β-CyD (mM)
HP-β-CyD (mM)
C
D
### Chart: Jurkat
| Category | G1 | S | G2/M |
|---|---|---|---|
| 0 | 45.166666666666664 | 33.1 | 15.1 |
| 5 | 46.23333333333333 | 32.26666666666667 | 14.5 |
| 10 | 21.400000000000002 | 34.06666666666667 | 29.5 |
### Chart: MOLT-4
| Category | G1 | S | G2/M |
|---|---|---|---|
| 0 | 51.699999999999996 | 29.366666666666664 | 5.789999999999999 |
| 5 | 47.199999999999996 | 32.800000000000004 | 5.489999999999999 |
| 10 | 28.833333333333332 | 39.86666666666667 | 16.766666666666666 |(%)
(%)
HP-β-CyD (mM)
HP-β-CyD (mM)
