# Supplementary figures and images for: 2-Hydroxypropyl-β-Cyclodextrin Acts as a Novel Anticancer Agent
Source: PLoS One. 2015 Nov 4;10(11):e0141946. doi: 10.1371/journal.pone.0141946 (PMC4633159; doi:10.1371/journal.pone.0141946)

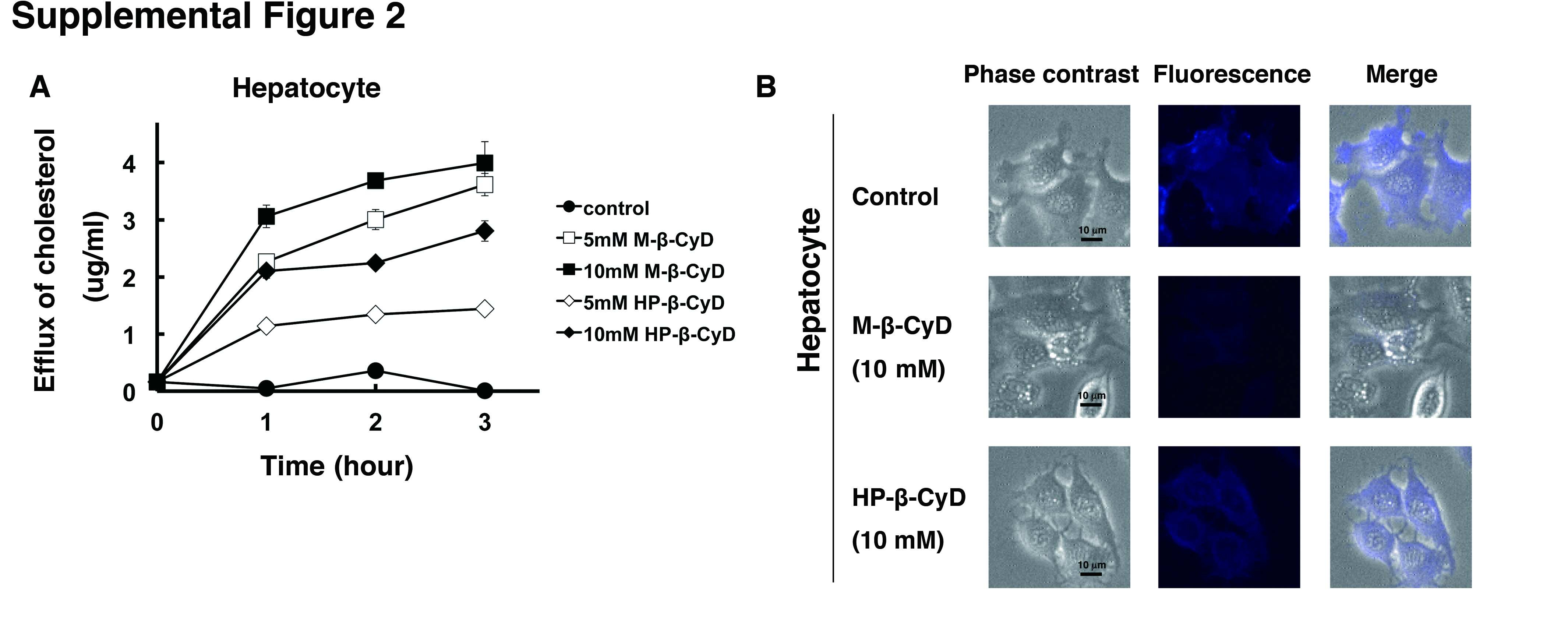

Supplement: S2 Fig — (A) Hepatocytes (1 × 10^7^ cells) were incubated in HBSS (pH 7.4) with or without HP-β-CyD or M-β-CyD (0, 5, 10 mM) for 1 hour. The concentration of cholesterol in HBSS were determined by Cholesterol E-test Wako®. (B) Image of filipin staining for hepatocytes. Primary hepatocytes were incubated with M-β-CyD (10 mM) or HP-β-CyD (10 mM) for 1 hour. Then, cells were treated with Filipin solution, and were scanned with a fluorescence microscope. (TIF) [file pone.0141946.s002.tif]

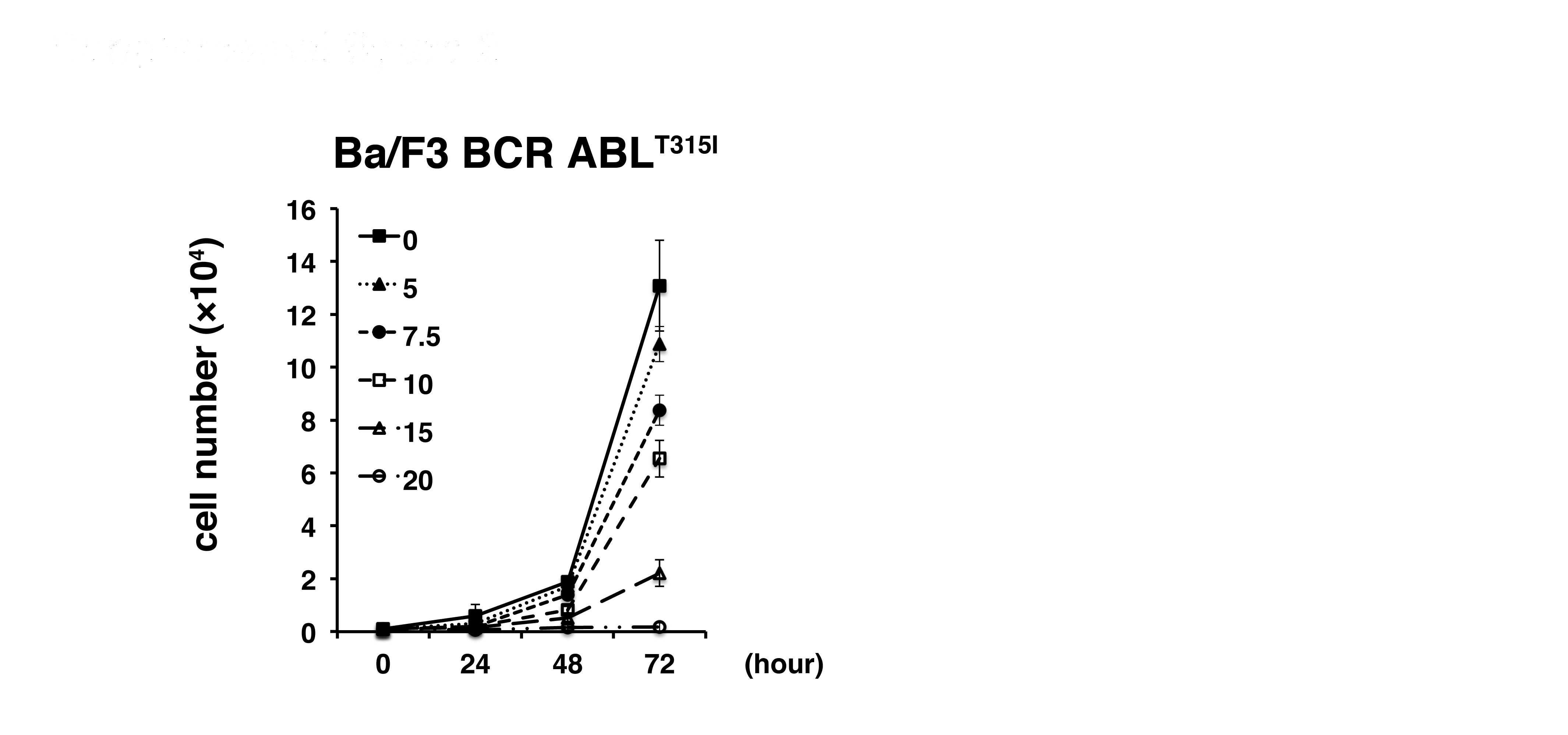

Supplement: S3 Fig — Ba/F3 BCR-ABLT315I cells were exposed to 0 mM (■), 5 mM (▲), 7.5 mM (●), 10 mM (□), 15 mM (△), and 20 mM (○) HP-β-CyD. Viable cells were counted by a trypan blue dye exclusion method. Data are the mean ± SD of three independent experiments. (TIF) [file pone.0141946.s003.tif]

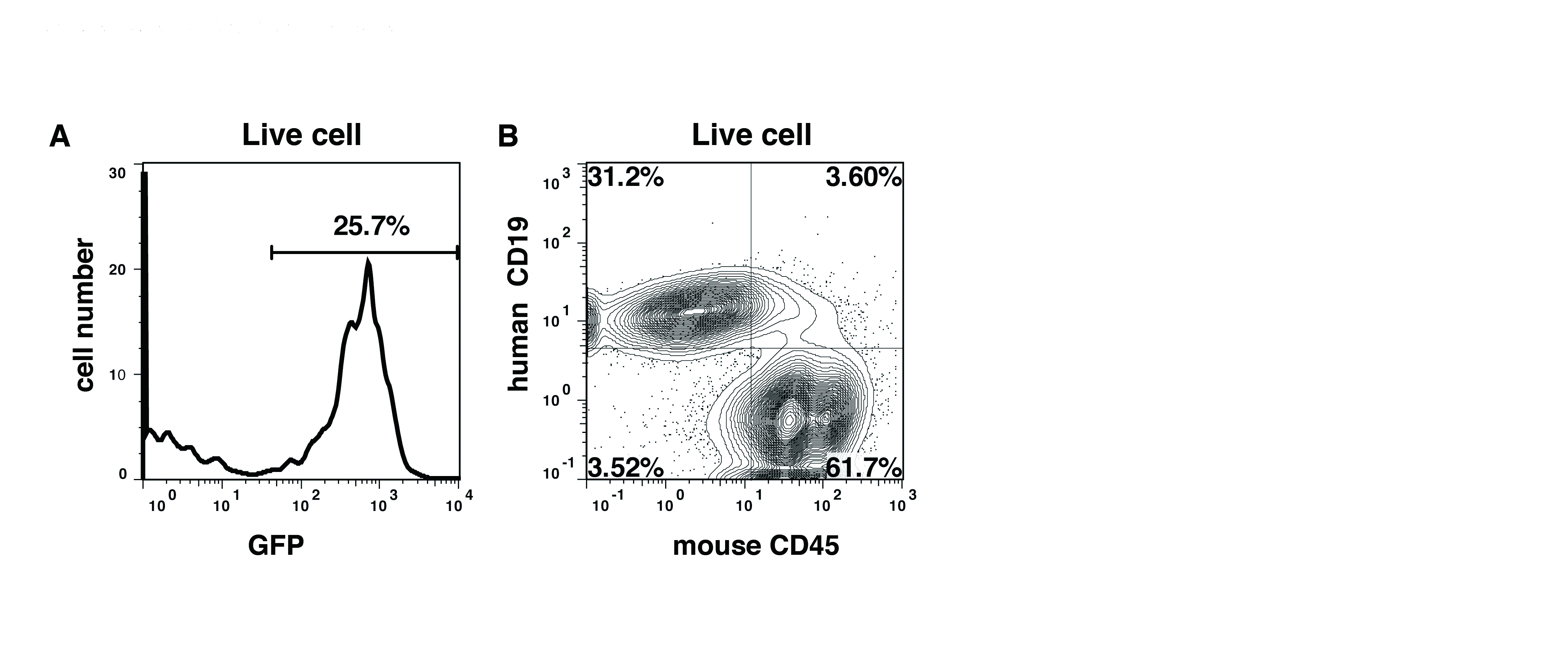

Supplement: S4 Fig — (A) Flow cytometric histogram of EGFP-positive BM cells from untreated nude mice that received EGFP+ Ba/F3 BCR-ABLWT cells. (B) Representative FACS plot of BV173 cell-transplanted NOD/SCID mice. BM cells of NOD/SCID mice were analyzed by FACS 4 weeks after BV173 cell transplantation using an anti-human CD19 antibody and anti-mouse CD45 antibody. (TIF) [file pone.0141946.s004.tif]
