# Supplementary material for: 2-Hydroxypropyl-β-Cyclodextrin Acts as a Novel Anticancer Agent
Source: PLoS One. 2015 Nov 4;10(11):e0141946. doi: 10.1371/journal.pone.0141946 (PMC4633159; doi:10.1371/journal.pone.0141946)
Supplement: S5 Fig — (A-B) K562/HA cells and KCL22/HA cells were treated with 0, 5 mM, 10 mM, 15 mM HP-β-CyD, respectively. After 24 hours of culture, cells were collected and stained with Annexin V and 7-AAD. (A) Percentage of Annexin V-positive K562/HA cells after culture with HP-β-CyD for 24 hours. Data are the mean ± SD of three independent experiments. (B) Percentage of Annexin V-positive KCL22 cells after culture with HP-β-CyD for 24 hours. Data are the mean ± SD of three independent experiments. **P < 0.01. (C-D) HP-β-CyD causes cell-cycle arrest in hypoxia-adapted leukemic cells. K562/HA and KCL22/HA cells were treated with the indicated concentration of HP-β-CyD for 12 hours, then flow cytometric analysis of PI-stained nuclei was performed. (C) The percentage of cells in G0/G1, S, or G2/M phase was assessed in viable K562/HA cells. White: G1-phase, gray: S-phase, black: G2/M-phase. (D) The percentage of cells in G0/G1, S, or G2/M phase was assessed in viable KCL22/HA cells. White: G1-phase, gray: S-phase, black: G2/M-phase. Data are the mean ± SD of three independent experiments. (PPTX) [file pone.0141946.s005.pptx]

## Slide 1
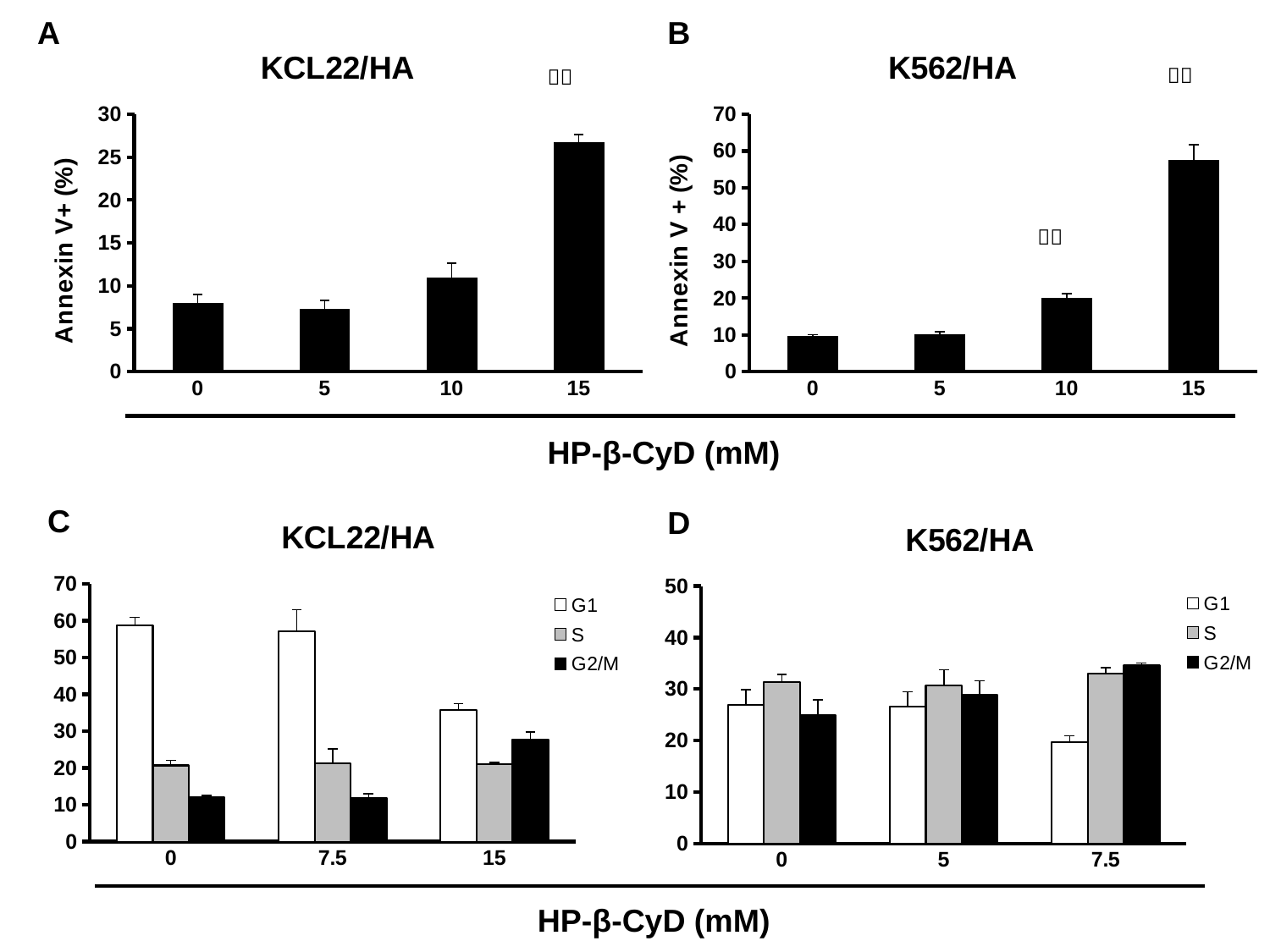

A
B
### Chart: KCL22/HA
| Category | 24hr |
|---|---|
| 0 | 7.989999999999999 |
| 5 | 7.363333333333333 |
| 10 | 10.950000000000001 |
| 15 | 26.700000000000003 |
### Chart: K562/HA
| Category | 24hr |
|---|---|
| 0 | 9.773333333333333 |
| 5 | 10.076666666666666 |
| 10 | 20.09333333333333 |
| 15 | 57.5 |＊＊
＊＊
＊＊
HP-β-CyD (mM)
C
### Chart: KCL22/HA
| Category | G1 | S | G2/M |
|---|---|---|---|
| 0 | 58.73333333333333 | 20.7 | 12.1 |
| 7.5 | 57.06666666666666 | 21.333333333333332 | 11.833333333333334 |
| 15 | 35.7 | 20.95 | 27.633333333333336 |D
### Chart: K562/HA
| Category | G1 | S | G2/M |
|---|---|---|---|
| 0 | 26.866666666666664 | 31.366666666666664 | 24.900000000000002 |
| 5 | 26.633333333333336 | 30.733333333333334 | 28.900000000000002 |
| 7.5 | 19.666666666666668 | 32.93333333333333 | 34.56666666666667 |HP-β-CyD (mM)
