# Supplementary material for: 2-Hydroxypropyl-β-Cyclodextrin Acts as a Novel Anticancer Agent
Source: PLoS One. 2015 Nov 4;10(11):e0141946. doi: 10.1371/journal.pone.0141946 (PMC4633159; doi:10.1371/journal.pone.0141946)
Supplement: S1 Table — Data from CBC counts of peripheral blood collected by retro-orbital bleeding of vehicle-, and HP-β-CyD-injected nude mice. Data are mean ± SD of three mice. (DOCX) [file pone.0141946.s006.docx]

**S1 Table. Red blood cell count in HP-β-CyD-injected nude mice**

Data from CBC counts of peripheral blood collected by retro-orbital bleeding of vehicle-, and HP-β-CyD-injected nude mice. Data are mean ± SD of three mice.
