# Supplementary material for: 2-Hydroxypropyl-β-Cyclodextrin Acts as a Novel Anticancer Agent
Source: PLoS One. 2015 Nov 4;10(11):e0141946. doi: 10.1371/journal.pone.0141946 (PMC4633159; doi:10.1371/journal.pone.0141946)
Supplement: S2 Table — Data from CBC counts of peripheral blood collected by retro-orbital bleeding of vehicle-injected, and NOD/SCID mice that received 50 mM HP-β-CyD administration for 7 weeks. Data are average of two mice. (DOCX) [file pone.0141946.s007.docx]

**S2 Table. Red blood cell count in HP-β-CyD-injected NOD/SCID mice**

Data from CBC counts of peripheral blood collected by retro-orbital bleeding of vehicle-injected, and NOD/SCID mice that received 50 mM HP-β-CyD administration for 7 weeks. Data are average of two mice.
